# A new era in the discovery of *de novo* mutations underlying human genetic disease

**DOI:** 10.1186/1479-7364-6-27

**Published:** 2012-12-12

**Authors:** Chee-Seng Ku, Vasilis Vasiliou, David N Cooper

**Affiliations:** 1 Department of Medical Epidemiology and Biostatistics, Karolinska Institutet, Nobels väg 12B, Stockholm, SE-171 77, Sweden; 2 Department of Pharmaceutical Sciences, University of Colorado Anschutz Medical Campus, 12850 East Montview Blvd, Aurora, CO, 80045, USA; 3 Institute of Medical Genetics, Cardiff University, Heath Park, Cardiff, Cardiff, CF14 4XN, UK

**Keywords:** *de novo* mutation, Exome sequencing, Human disease

## Editorial text

Germline mutations arise anew during meiosis in every generation. Such spontaneously occurring genetic alterations are termed *de novo* mutations and serve to describe those heritable mutations that neither parent possessed or transmitted. Thus, *de novo* mutations denote mutations that arose in the gametes of the parents as distinct from post-zygotic somatic mutations that arise during embryonic development. Studies of *de novo* mutations in the human genome have been very challenging owing to past technological limitations. However, the advent of high-through put next-generation sequencing (NGS) technologies has ushered in a new era in the study of *de novo* mutations. Whole-genome sequencing (WGS) and whole-exome sequencing (WES) can now be performed on parent-offspring trios to identify *de novo* point mutations in the entire genome or within protein-coding regions, respectively [1-3]. Although genome-wide *de novo* copy number variants (CNVs) were previously studied using microarrays [4-7], the study of *de novo* point mutations or single nucleotide variants (SNVs) required the advent of large-scale sequencing, which was not feasible using Sanger sequencing [1-3]. As with inherited mutations, *de novo* mutations range in size from point mutations and small indels of multiple bases to much larger CNVs and structural rearrangements [8]. In this article, we focus on *de novo* point mutations/SNVs and their implications for our understanding of the etiology of common complex diseases as well as rare Mendelian diseases.

The availability of NGS has allowed first estimations of the human genome mutation rate as well as a glimpse of the spatial distribution of *de novo* point mutations in the human genome and their association with heritable diseases [1-3,9-13]. Recent studies have estimated the per-generation mutation rate in humans as approximately 10^−8^. For example, sequencing the complete genomes from two parent-offspring trios (from the HapMap CEU and YRI, to >22-fold mapped depth) identified a total of 49 and 35 germline *de novo* point mutations in the two trios, respectively [2]. Similarly, an average *de novo* mutation rate of 1.20 × 10^−8^ per nucleotide per generation was also reported by sequencing the entire genomes of 78 Icelandic parent-offspring trios [3]. Interestingly, the diversity in the mutation rate of SNVs was found to be strongly influenced by the age of the father when the child was conceived (the impact of paternal age corresponds to about two mutations per year). These findings have highlighted the likely importance of the father's age for the risk of those diseases which appear to be strongly affected by *de novo* mutations, e.g., schizophrenia and autism (see below) [3]. However, the mutation rate is critically dependent upon the type of mutation being considered, as the CNV mutation rate is somewhat higher than the single base-pair substitution mutation rate [14].

Owing to technical limitations, the contribution of *de novo* point mutations to human genetic diseases (both common complex disease and rare Mendelian disorder) has remained largely unexplored. *De novo* point mutations are nevertheless likely to have severe biological or phenotypic consequences when they impact functionally important nucleotides in the genome (for example, nonsense mutations or missense mutations in evolutionarily conserved regions). However, only a relatively small proportion of the *de novo* point mutations occurring at each meiosis will alter functionally important nucleotides [1-3]. Although *de novo* CNVs have been shown, using whole-genome genotyping arrays, to be associated with several common neurodevelopmental diseases such as schizophrenia and autism [4-7], it was not until very recently that *de novo* point mutations were also implicated in the etiology of schizophrenia, autism, and mental retardation through the WES of affected trios [9-12,15-17]. In similar vein, *de novo* point mutations have also been found to be responsible for sporadic cases of various rare dominant Mendelian disorders such as Kabuki syndrome, Schinzel-Giedion syndrome, and Bohring-Opitz syndrome [18-20]. These Mendelian disorders are also characterized by multiple neurodevelopmental defects; thus, for example, Bohring-Opitz syndrome is a clinically recognizable syndrome characterized by severe intellectual disability, distinctive facial features, and multiple congenital malformations [20].

It has been postulated that the occurrence of *de novo* mutations might explain why diseases characterized by dramatically reduced fecundity such as mental retardation and schizophrenia nevertheless remain frequent in the population. If this were indeed to be the case, the *de novo* mutations occurring in sporadic cases of these diseases would serve to replenish the number of highly penetrant disease mutations in every generation despite the continual negative selection. Indeed, *de novo* CNVs are a known cause of schizophrenia, autism, and mental retardation [21,22]. In addition, *de novo* point mutations have also been identified in schizophrenia through the WES of affected case-parent trios. In the exomes of 53 sporadic schizophrenia cases (with no history of the disease in a first- or second-degree relative), 22 unaffected controls and their parents were sequenced which led to the identification of a total of 34 *de novo* point mutations/SNVs and 4 *de novo* indels in the affected trios. However, it is noteworthy that some *de novo* SNVs (four non-synonymous and three synonymous) were also identified in the control subjects. This suggests that identifying the pathogenic and/or deleterious *de novo* mutations from a non-disease-associated mutational background may be a tricky task [11]. In a parallel development, *de novo* mutations were also found to be enriched in autism spectrum disorder (ASD)/autism [15-17]. A large WES study of 238 families from a comprehensively phenotyped ASD cohort comprising pedigrees with two unaffected parents and an affected proband (and in 200 families, an unaffected sibling) found that highly disruptive (nonsense and splice-site) *de novo* point mutations in brain-expressed genes were associated with ASDs and that these mutations carried large effects. *De novo* point mutations have also been identified in individuals with unexplained mental retardation [9]. These findings provide further support for the likely importance of *de novo* mutations in the etiology of these complex neurodevelopmental disorders.

*De novo* heterozygous point mutations are also believed to be a common cause of sporadic cases of those rare Mendelian diseases that are characterized by multiple congenital malformations or anomalies, developmental delay, and mental retardation or intellectual disability such as Schinzel-Giedion syndrome and Bohring-Opitz syndrome, the genetic basis of which had previously remained elusive [19,20]. These disease-causing *de novo* mutations were undetected by linkage analysis because they were not transmitted between the generations. Although the conventional gene-centric approach of limited Sanger sequencing is capable of identifying *de novo* point mutations, this method cannot realistically be scaled up to encompass the entire genome, exome, or even all genes on a chromosomal arm. Further, it is a hypothesis-driven approach whereby the selection of candidate genes is highly dependent upon an *a priori* hypothesis which is in turn constrained by our still limited knowledge of the biological pathways that characterize the diseases in question. By contrast, WES constitutes a powerful and robust tool which can be used to unravel *de novo* point mutations by screening the entire coding region of the genome (i.e., all the exons) of the affected parent-offspring trios. The first success in identifying novel *de novo* mutations for rare diseases came with the identification of the *MLL2* gene underlying Kabuki syndrome, even although this study did not adopt a trio design [18]. Instead, the exomes of unrelated probands from different families were sequenced in order to identify the causal genes, and in families where parental DNA was available, the mutations were confirmed to be *de novo* in some of them [18]. However, similar success was also obtained in studies that applied WES to affected trios for various rare diseases. Thus, *de novo* mutations in the *EZH2* gene underlying Weaver syndrome [23] and in the *ACTB* and *ACTG1* genes underlying Baraitser-Winter syndrome were identified by means of WES of trios [24].

The advantage of applying WES to parent-case trios is that it narrows down the list of potential candidate disease mutations very considerably because of the limited number of *de novo point* mutations to be expected within protein-coding sequences. This is of course especially useful for disorders in which *de novo* mutations are strongly suspected, e.g., Baraitser-Winter syndrome (a rare but now well-defined developmental disorder). No familial recurrence or consanguinity has ever been observed in families affected with this syndrome; hence, the genetic basis of Baraitser-Winter syndrome was always likely to be due to the occurrence of *de novo* point mutations (as no pathogenic CNVs had been detected using microarrays) [24]. WES would be sufficient to identify the mutations underlying most rare Mendelian disorders as long as the causative mutations reside within the coding regions. In contrast to WGS, WES is more cost-effective and analytically less challenging. However, some studies have also applied the WGS approach, for example, to a family quartet affected by a sporadic case of severe epileptic encephalopathy. WGS of the family, containing an affected proband and her unaffected parents and sibling, revealed a *de novo* heterozygous missense mutation in *SCN8A*[25].

Although numerous *de novo* point mutations have now been identified which are responsible for rare disorders, it remains unclear what constitutes evidence for causality. It is both difficult and challenging to establish the causative role of newly identified mutations, even for *de novo* events. Co-segregation of the putative causal mutation with a disease phenotype in large pedigrees might provide strong genetic evidence of causality, but this would not be applicable in the case of *de novo* mutations which are, by definition, not transmitted through the generations. Therefore, for a newly identified putative pathological mutation which appears to have occurred *de novo*, further screening of additional cases is invariably required. Detection of recurrent mutation (or identical mutations) or different mutations in the same gene in additional cases and their absence in healthy individuals constitutes strong evidence of causality. However, it might be quite difficult, in the context of extremely rare disorders, to find additional cases with which to validate the newly identified *de novo* mutation. Although challenging, further validation in additional samples has generally been successful in recent WES studies of rare disorders. Most notably, after the discovery of *de novo* point mutations in *ACTG1* and *ACTB* by WES of three parent-case trios with Baraitser-Winter syndrome, the study used Sanger sequencing to screen the coding sequence of both genes in 15 additional affected individuals and was successful in detecting pathogenic mutations in one or other of these genes in all subjects studied [24]. These mutations were then shown to have occurred *de novo* in all 11 subjects from whom parental DNA was available. Moreover, further validation in normal controls showed that none of the mutations identified in Baraitser-Winter syndrome were present in several large control datasets [24]. As an alternative, molecular functional studies to confirm the functional significance of newly identified *de novo* mutations could be considered confirmatory if and when the mutated gene had a clear role in conferring a well-defined molecular pathology.

The studies of *de novo* mutations that have been performed so far have adopted the WES approach. However, the current shortcoming of WES lies in its necessary focus on coding regions. Hence, the clinicobiological importance of *de novo* mutations in non-coding regions remains unclear. Although very challenging, WGS eventually promises to permit the comprehensive analysis of *de novo* mutations (and other inherited variants) in the human genome in collections of case-parent trios affected by various heritable diseases. Since a considerable number of inherited mutations for various diseases have now been found in non-coding regions [26], it is reasonable to suppose that this could also pertain in the context of *de novo* mutations within non-coding regions. This in turn supports the likely efficacy of a strategy of adopting WGS to ascertain the full impact of *de novo* mutations in human genetic disease and to study the molecular characteristics of such lesions. The collection of a large number of such trios would be important in this context. The discovery to date of *de novo* mutations underlying common complex diseases as well as rare Mendelian disorders is likely to represent only the tip of the iceberg; more discoveries would be anticipated in the near future.

## Competing interests

The authors declared that they have no competing interests.

## Authors’ contributions

CSK and VV contributed to the conceptualization of this article. CSK and DNC contributed to the writing of this article. VV contributed to proofreading. CSK and DNC approved the final manuscript. All authors read and approved the final manuscript.

## References

[B1] Roach JC Glusman G Smit AF Huff CD Hubley R Shannon PT Rowen L Pant KP Goodman N Bamshad M Shendure J Drmanac R Jorde LB Hood L Galas DJ Analysis of genetic inheritance in a family quartet by whole-genome sequencing Science 2010 328 636 639 20220176 10.1126/science.1186802 PMC3037280

[B2] Conrad DF Keebler JE DePristo MA Lindsay SJ Zhang Y Casals F Idaghdour Y Hartl CL Torroja C Garimella KV Zilversmit M Cartwright R Rouleau GA Daly M Stone EA Hurles ME Awadalla P Genomes P Variation in genome-wide mutation rates within and between human families Nat Genet 2011 43 712 714 21666693 10.1038/ng.862 PMC3322360

[B3] Kong A Frigge ML Masson G Besenbacher S Sulem P Magnusson G Gudjonsson SA Sigurdsson A Jonasdottir A Jonasdottir A Wong WS Sigurdsson G Walters GB Steinberg S Helgason H Thorleifsson G Gudbjartsson DF Helgason A Magnusson OT Thorsteinsdottir U Stefansson K Rate of de novo mutations and the importance of father's age to disease risk Nature 2012 488 471 475 22914163 10.1038/nature11396 PMC3548427

[B4] Xu B Roos JL Levy S van Rensburg EJ Gogos JA Karayiorgou M Strong association of de novo copy number mutations with sporadic schizophrenia Nat Genet 2008 40 880 885 18511947 10.1038/ng.162

[B5] Rees E Moskvina V Owen MJ O'Donovan MC Kirov G De novo rates and selection of schizophrenia-associated copy number variants Biol Psychiatry 2011 70 1109 1114 21855053 10.1016/j.biopsych.2011.07.011

[B6] Kirov G Pocklington AJ Holmans P Ivanov D Ikeda M Ruderfer D Moran J Chambert K Toncheva D Georgieva L Grozeva D Fjodorova M Wollerton R Rees E Nikolov I van de Lagemaat LN Bayes A Fernandez E Olason PI Bottcher Y Komiyama NH Collins MO Choudhary J Stefansson K Stefansson H Grant SG Purcell S Sklar P O'Donovan MC Owen MJ De novo CNV analysis implicates specific abnormalities of postsynaptic signalling complexes in the pathogenesis of schizophrenia Mol Psychiatry 2012 17 142 153 22083728 10.1038/mp.2011.154 PMC3603134

[B7] Sanders SJ Ercan-Sencicek AG Hus V Luo R Murtha MT Moreno-De-Luca D Chu SH Moreau MP Gupta AR Thomson SA Mason CE Bilguvar K Celestino-Soper PB Choi M Crawford EL Davis L Wright NR Dhodapkar RM DiCola M DiLullo NM Fernandez TV Fielding-Singh V Fishman DO Frahm S Garagaloyan R Goh GS Kammela S Klei L Lowe JK Lund SC Multiple recurrent de novo CNVs, including duplications of the 7q11.23 Williams syndrome region, are strongly associated with autism Neuron 2011 70 863 885 21658581 10.1016/j.neuron.2011.05.002 PMC3939065

[B8] Cooper DN Bacolla A Ferec C Vasquez KM Kehrer-Sawatzki H Chen JM On the sequence-directed nature of human gene mutation: the role of genomic architecture and the local DNA sequence environment in mediating gene mutations underlying human inherited disease Hum Mutat 2011 32 1075 1099 21853507 10.1002/humu.21557 PMC3177966

[B9] Vissers LE de Ligt J Gilissen C Janssen I Steehouwer M de Vries P van Lier B Arts P Wieskamp N del Rosario M van Bon BW Hoischen A de Vries BB Brunner HG Veltman JA A de novo paradigm for mental retardation Nat Genet 2010 42 1109 1112 21076407 10.1038/ng.712

[B10] O'Roak BJ Deriziotis P Lee C Vives L Schwartz JJ Girirajan S Karakoc E Mackenzie AP Ng SB Baker C Rieder MJ Nickerson DA Bernier R Fisher SE Shendure J Eichler EE Exome sequencing in sporadic autism spectrum disorders identifies severe de novo mutations Nat Genet 2011 43 585 589 21572417 10.1038/ng.835 PMC3115696

[B11] Xu B Roos JL Dexheimer P Boone B Plummer B Levy S Gogos JA Karayiorgou M Exome sequencing supports a de novo mutational paradigm for schizophrenia Nat Genet 2011 43 864 868 21822266 10.1038/ng.902 PMC3196550

[B12] Girard SL Gauthier J Noreau A Xiong L Zhou S Jouan L Dionne-Laporte A Spiegelman D Henrion E Diallo O Thibodeau P Bachand I Bao JY Tong AH Lin CH Millet B Jaafari N Joober R Dion PA Lok S Krebs MO Rouleau GA Increased exonic de novo mutation rate in individuals with schizophrenia Nat Genet 2011 43 860 863 21743468 10.1038/ng.886

[B13] Scally A Durbin R Revising the human mutation rate: implications for understanding human evolution Nat Rev Genet 2012 13 745 753 22965354 10.1038/nrg3295

[B14] Itsara A Wu H Smith JD Nickerson DA Romieu I London SJ Eichler EE De novo rates and selection of large copy number variation Genome Res 2010 20 1469 1481 20841430 10.1101/gr.107680.110 PMC2963811

[B15] Neale BM Kou Y Liu L Ma'ayan A Samocha KE Sabo A Lin CF Stevens C Wang LS Makarov V Polak P Yoon S Maguire J Crawford EL Campbell NG Geller ET Valladares O Schafer C Liu H Zhao T Cai G Lihm J Dannenfelser R Jabado O Peralta Z Nagaswamy U Muzny D Reid JG Newsham I Wu Y Patterns and rates of exonic de novo mutations in autism spectrum disorders Nature 2012 485 242 245 22495311 10.1038/nature11011 PMC3613847

[B16] Sanders SJ Murtha MT Gupta AR Murdoch JD Raubeson MJ Willsey AJ Ercan-Sencicek AG DiLullo NM Parikshak NN Stein JL Walker MF Ober GT Teran NA Song Y El-Fishawy P Murtha RC Choi M Overton JD Bjornson RD Carriero NJ Meyer KA Bilguvar K Mane SM Sestan N Lifton RP Gunel M Roeder K Geschwind DH Devlin B State MW De novo mutations revealed by whole-exome sequencing are strongly associated with autism Nature 2012 485 237 241 22495306 10.1038/nature10945 PMC3667984

[B17] O'Roak BJ Vives L Girirajan S Karakoc E Krumm N Coe BP Levy R Ko A Lee C Smith JD Turner EH Stanaway IB Vernot B Malig M Baker C Reilly B Akey JM Borenstein E Rieder MJ Nickerson DA Bernier R Shendure J Eichler EE Sporadic autism exomes reveal a highly interconnected protein network of de novo mutations Nature 2012 485 246 250 22495309 10.1038/nature10989 PMC3350576

[B18] Ng SB Bigham AW Buckingham KJ Hannibal MC McMillin MJ Gildersleeve HI Beck AE Tabor HK Cooper GM Mefford HC Lee C Turner EH Smith JD Rieder MJ Yoshiura K Matsumoto N Ohta T Niikawa N Nickerson DA Bamshad MJ Shendure J Exome sequencing identifies MLL2 mutations as a cause of Kabuki syndrome Nat Genet 2010 42 790 793 20711175 10.1038/ng.646 PMC2930028

[B19] Hoischen A van Bon BW Gilissen C Arts P van Lier B Steehouwer M de Vries P de Reuver R Wieskamp N Mortier G Devriendt K Amorim MZ Revencu N Kidd A Barbosa M Turner A Smith J Oley C Henderson A Hayes IM Thompson EM Brunner HG de Vries BB Veltman JA De novo mutations of SETBP1 cause Schinzel-Giedion syndrome Nat Genet 2010 42 483 485 20436468 10.1038/ng.581

[B20] Hoischen A van Bon BW Rodriguez-Santiago B Gilissen C Vissers LE de Vries P Janssen I van Lier B Hastings R Smithson SF Newbury-Ecob R Kjaergaard S Goodship J McGowan R Bartholdi D Rauch A Peippo M Cobben JM Wieczorek D Gillessen-Kaesbach G Veltman JA Brunner HG de Vries BB De novo nonsense mutations in ASXL1 cause Bohring-Opitz syndrome Nat Genet 2011 43 729 731 21706002 10.1038/ng.868

[B21] Malhotra D Sebat J CNVs: harbingers of a rare variant revolution in psychiatric genetics Cell 2012 148 1223 1241 22424231 10.1016/j.cell.2012.02.039 PMC3351385

[B22] Rodriguez-Murillo L Gogos JA Karayiorgou M The genetic architecture of schizophrenia: new mutations and emerging paradigms Annu Rev Med 2012 63 63 80 22034867 10.1146/annurev-med-072010-091100

[B23] Gibson WT Hood RL Zhan SH Bulman DE Fejes AP Moore R Mungall AJ Eydoux P Babul-Hirji R An J Marra MA Consortium FC Chitayat D Boycott KM Weaver DD Jones SJ Mutations in EZH2 cause Weaver syndrome Am J Hum Genet 2012 90 110 118 22177091 10.1016/j.ajhg.2011.11.018 PMC3257956

[B24] Riviere JB van Bon BW Hoischen A Kholmanskikh SS O'Roak BJ Gilissen C Gijsen S Sullivan CT Christian SL Abdul-Rahman OA Atkin JF Chassaing N Drouin-Garraud V Fry AE Fryns JP Gripp KW Kempers M Kleefstra T Mancini GM Nowaczyk MJ van Ravenswaaij-Arts CM Roscioli T Marble M Rosenfeld JA Siu VM de Vries BB Shendure J Verloes A Veltman JA Brunner HG De novo mutations in the actin genes ACTB and ACTG1 cause Baraitser-Winter syndrome Nat Genet 2012 44 440 444 22366783 10.1038/ng.1091 PMC3677859

[B25] Veeramah KR O'Brien JE Meisler MH Cheng X Dib-Hajj SD Waxman SG Talwar D Girirajan S Eichler EE Restifo LL Erickson RP Hammer MF De novo pathogenic SCN8A mutation identified by whole-genome sequencing of a family quartet affected by infantile epileptic encephalopathy and SUDEP Am J Hum Genet 2012 90 502 510 22365152 10.1016/j.ajhg.2012.01.006 PMC3309181

[B26] Cooper DN Chen JM Ball EV Howells K Mort M Phillips AD Chuzhanova N Krawczak M Kehrer-Sawatzki H Stenson PD Genes, mutations, and human inherited disease at the dawn of the age of personalized genomics Hum Mutat 2010 31 631 655 20506564 10.1002/humu.21260

